# Strained contacts with the cell membrane may influence ligand affinity to G protein coupled receptors: a case of free fatty acid receptor 1 agonists

**DOI:** 10.1080/14756366.2021.1955874

**Published:** 2021-07-22

**Authors:** Alexey Lukin, Anna Bakholdina, Mikhail Chudinov, Oleksandra Onopchenko, Elena Zhuravel, Sergey Zozulya, Maxim Gureev, Mikhail Krasavin

**Affiliations:** a Lomonosov Institute of Fine Chemical Technologies, MIREA – Russian Technological University, Moscow, Russian Federation; b Enamine Ltd., Kyiv, Ukraine; c Taras Shevchenko National University, Kyiv, Ukraine; d Digital Biodesign and Personalized Healthcare Research Center, Sechenov First Moscow State Medical University, Moscow, Russian Federation; e Saint Petersburg State University, Saint Petersburg, Russian Federation

**Keywords:** G protein-coupled receptor, free fatty acid receptor 1 agonist, docking score, phospholipid cell membrane bilayer, strained ligand interactions with cell membrane

## Abstract

A set of 1,3,4-thiadiazole-2-carboxamides bearing a substituted biphenyl in the amide portion was synthesised and tested for agonistic activity towards free fatty acid receptor 1 (FFA1). The observed activity trends were impossible to rationalised based solely on the docking energy scores of Glide SP. On the contrary, when the phospholipid cell membrane bilayer was reconstructed around FFA1, it became apparent that inactive compounds displayed significant strained contacts with the membrane while for active compounds the strain was noticeably lower. These findings justify using the improved docking protocol for modelling GPCR-ligand interactions which uses the crystal structure of the receptor and a reconstructed portion of a cell membrane.

## Graphical Abstract

**Figure:**
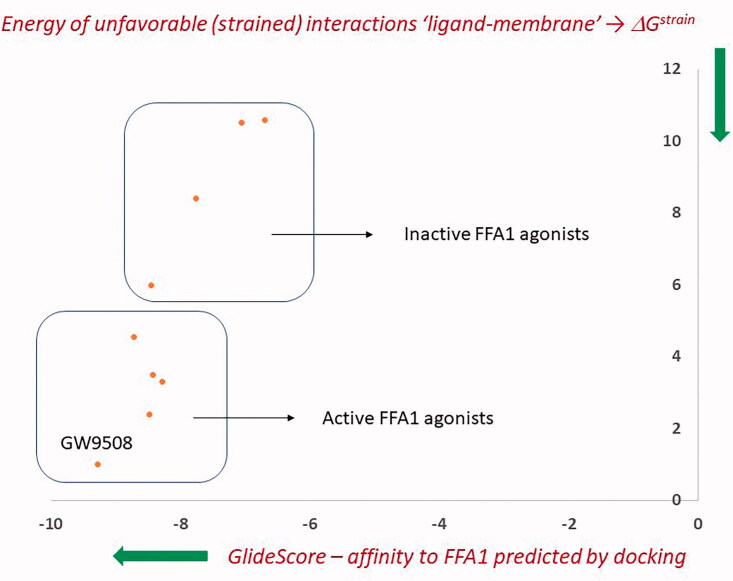

## 1. Introduction

Small molecule agents that act as agonists for free fatty acid receptor 1 (FFA1, referred to as GPR40 before it was de-orphaned in 2003) is a promising class of drugs to treat type 2 diabetes mellitus (T2DM) which holds no risk of causing the development of hypoglycaemia^1^. Under normal glycaemia, expression of FFA1 (mostly in the islets of Langehans of the pancreas) is low. Once the levels of glucose go up, for example, in the diabetic state, so do the expression levels of FFA1. Administration of agonists at this point was shown to lower the levels of glucose and, consequently, leads to internalisation and downregulation of FFA1^2^. Therefore, the decade between 2003 and 2013 was marked by intensified research efforts aimed at bringing FFA1 into the clinic for the treatment of T2DM^3^. Unfortunately, the frontrunner clinical candidate, first-in-class agent fasiglifam (TAK-875) developed by Takeda was discontinued in phase III clinical trials due to idiosyncratic liver toxicity notes in large patient populations^4^. This dramatic setback caused the majority of Takeda’s competition to exit the field. At the time of writing this manuscript no clinical studies of agents acting as FFA1 agonists were underway. However, the efficacy results obtained in the course of TAK-875 phase II study provided a solid proof-of-principle for the new therapeutic approach^5^. Hence, putting new agents from various chemical classes in FFA1 agonist development pipeline – focussing on overcoming the liver toxicity^6^ – is a worthy undertaking.

**Figure 1.**
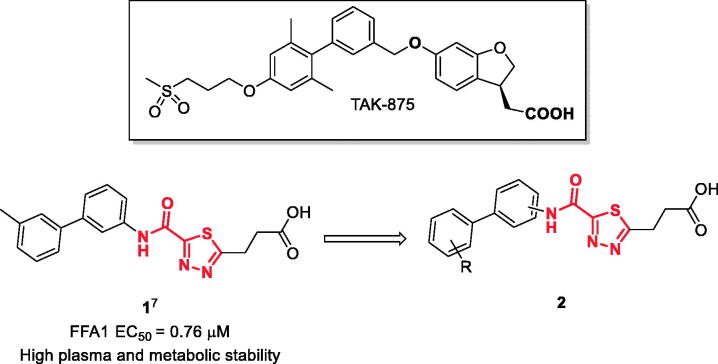
Structure of TAK-875, the earlier reported 1,3,4-thiadiazole-2-carboxamide lead molecule **1** and the general structure of biphenyl analogs **2** explored in this work.

Earlier, we reported on the discovery of novel FFA1 agonists based on 1,3,4-thiadiazole-2-carboxamide scaffold^7^. Out of this series, 4-(3-methyl)phenyl anilide **1** emerged as a potent (FFA1 EC_50_ 0.76 µM) lead with high stability in plasma and in the presence of liver microsomes. Encouraged by this result, we proceeded to explore other substituted biphenyls in the carboxamide portion of the lead series and synthesised a number of new derivatives **2** belonging to this class (Figure 1).

Unfortunately, evaluation of agonistic potency of the newly synthesised compounds **2** revealed a disappointingly “flat” structure-activity relationships (SAR) profile and no breaking into the nanomolar activity range. Moreover, some of the drastic differences in the activity of structurally related analogs appeared inexplicable based on the general understanding of the small molecule binding to its protein target: small changes in the substitution pattern cause the activity towards FFA1 to be completely lost (*vide infra*). Hence, we set off to rationalise the observed potency trends by factors other than sheer affinity to the receptor. Mindful of the fact that FFA1 is a G-protein coupled receptors integrated into the phospholipid cell membrane bilayer in a heptahelical transmembrane architecture, we hypothesised that looking at the interaction of small molecule ligands with the entire protein-phospholipid membrane complex may provide a deeper understanding of what structural factors govern the affinity and functional potency of FFA1 agonists. Indeed, similarly to the cell membrane’s role in inducing an active conformation of certain GPCRs^8^, favourable or unfavourable (strained) interactions of GPCR ligands with the cell membrane may influence the observed functional response of the receptor to small-molecule modulation. Herein, we summarise our preliminary findings which support this hypothesis.

## 2. Materials and methods

### 2.1. Chemical syntheses – general

All reactions were conducted in oven-dried glassware in the atmosphere of nitrogen. Melting points were measured with a Buchi В-520 melting point apparatus and were not corrected. Analytical thin-layer chromatography was carried out on Silufol UV-254 silica gel plates using appropriate mixtures of ethyl acetate and hexane. Compounds were visualised with short-wavelength UV light. ^1^H NMR and ^13^C NMR spectra were recorded on Bruker MSL-300 spectrometers in DMSO-d6 using TMS as an internal standard. Mass spectra were recorded using Shimadzu LCMS-2020 system with electron-spray (ESI) ionisation. All and reagents and solvents were obtained from commercial sources and used without purification.

### 2.2. General procedure 1: preparation of compounds 3a–h (exemplified for compound 3a)

To chloroacetyl chloride (4.1 mmol, 0.47 g) in anhydrous toluene (30 ml) 4′-chlorobiphenyl-3-amine (3.9 mmol, 0.80 g) was added with stirring. The reaction mixture was heated at reflux for 2 h. The progress of the reaction was monitored using thin-layer chromatography and 30% ethyl acetate in petroleum ether as eluent. The reaction mixture was cooled to room temperature and left without stirring until the formation of the precipitate was complete. The resulting precipitate was filtered off and the filtrate was concentrated *in vacuo*. To finely ground sulphur (7.9 mmol, 0.25 g), suspended in *N,N*-dimethylformamide (5.0 ml), was added triethylamine (7.9 mmol, 1.10 ml) and morpholine (5.3 mmol, 0.46 ml). After 15 min stirring at room temperature, the residue obtained in the first chemical operation was added and the mixture was stirred at room temperature for 3 h. The progress of the reaction was monitored using thin-layer chromatography and 1% methanol in chloroform as eluent. The reaction mixture was poured into water. The resulting precipitate was filtered off, 20 ml of acetone was added to the resulting precipitate, and undissolved excess sulphur was filtered off. The organic phase was separated, dried over anhydrous sodium sulphate, filtered, and concentrated *in vacuo*. The residue was taken up in *N,N*-dimethylformamide (5 ml), and treated with hydazine hydrate (4.1 µmol, 2.00 ml). The progress of the reaction was monitored using thin-layer chromatography and 1% methanol in chloroform as eluent. 100 ml of water was added to the reaction mixture and the resulting solution was acidified with 5% hydrochloric acid solution to pH = 5. The precipitate formed was filtered off and the filtrate was collected and concentrated *in vacuo*. The product was obtained by crystallisation of the residue from diethyl ether.

#### 2.2.1. N-(4′-Chlorobiphenyl-3-yl)-2-hydrazino-2-thioxoacetamide (3a)

Yield 0.37 g (31%), m.p. 188–189°С. ^1^H NMR (300 MHz, DMSO) *δ* 10.34 (s, 1H), 8.07 (br.s, 1H), 7.88–7.80 (m, 1H), 7.69 (d, *J =* 8.5 Hz, 2H), 7.59–7.43 (m, 4H); ^13^C NMR (75 MHz, DMSO) *δ* 167.3, 158.1, 139.4, 138.6, 138.2, 132.6, 129.6, 129.0, 128.5, 122.8, 119.4, 118.5.

#### 2.2.2. N-(4′-Chlorobiphenyl-4-yl)-2-hydrazino-2-thioxoacetamide (3b)

Yield 0.37 g (31%), m.p. 215–216°С. ^1^H NMR (300 MHz, DMSO) *δ* 10.33 (br.s, 1H), 7.87 (d, *J =* 8.6 Hz, 2H), 7.75–7.65 (m, 4H), 7.50 (d, *J =* 8.5 Hz, 2H); ^13^C NMR (75 MHz, DMSO) *δ* 167.4, 158.0, 138.3, 137.3, 134.8, 132.1, 128.9, 128.2, 127.0, 120.6.

#### 2.2.3. 2-Hydrazino-2-thioxo-N-[4′-(trifluoromethoxy)biphenyl-3-yl]acetamide (3c)

Yield 0.37 g (33%), m.p. 162–163°С. ^1^H NMR (300 MHz, DMSO) *δ* 10.34 (s, 1H), 8.10–8.06 (m, 1H), 7.88–7.83 (m, 1H), 7.81–7.76 (m, 2H), 7.49–7.44 (m, 4H); ^13^C NMR (75 MHz, DMSO) *δ* 167.4, 158.1, 148.0 (q, *J =* 1.7 Hz), 139.3, 139.1, 138.1, 129.6, 128.6, 123.0, 121.4, 120.1 (q, *J =* 256.4 Hz), 119.5, 118.7.

#### 2.2.4. 2-Hydrazino-N-(4′-methoxybiphenyl-4-yl)-2-thioxoacetamide (3d)

Yield 0.38 g (31%), m.p. 205–206°С. ^1^H NMR (300 MHz, DMSO) *δ* 10.11 (br.s, *J =* 33.4 Hz, 1H), 7.65 (d, *J =* 8.5 Hz, 2H), 7.56–7.35 (m, 4H), 6.84 (d, *J =* 8.5 Hz, 2H), 3.62 (br.s, 3H); ^13^C NMR (75 MHz, DMSO) *δ* 167.5, 158.8, 158.7, 136.3, 136.0, 131.9, 127.5, 126.5, 120.6, 114.4, 55.2.

#### 2.2.5. 2-Hydrazino-2-thioxo-N-[3′-(trifluoromethyl)biphenyl-4-yl]acetamide (3e)

Yield 0.40 g (30%), m.p. 146–147°С. ^1^H NMR (300 MHz, DMSO) *δ* 10.36 (br.s, 1H), 8.02–7.96 (m, 2H), 7.91 (d, *J =* 8.7 Hz, 2H), 7.78 (d, *J =* 8.7 Hz, 2H), 7.72–7.67 (m, 2H); ^13^C NMR (75 MHz, DMSO) *δ* 167.6, 158.1, 140.5, 137.6, 134.5, 130.4, 130.1, 129.8 (q, *J =* 31.6 Hz), 127.4, 124.2 (q, *J =* 272.4 Hz), 123.8 (q, *J =* 3.6 Hz), 122.7 (q, *J =* 3.9 Hz), 120.6.

#### 2.2.6. 2-Hydrazino-2-thioxo-N-[3′-(trifluoromethyl)biphenyl-3-yl]acetamide (3f)

Yield 0.39 g (29%), m.p. 159–160°С. ^1^H NMR (300 MHz, DMSO) *δ* 10.37 (br.s, 1H), 8.15 (br.s, 1H), 8.04–7.88 (m, 3H), 7.78–7.67 (m, 2H), 7.58–7.46 (m, 2H); ^13^C NMR (75 MHz, DMSO) *δ* 167.2, 158.2, 140.8, 139.1, 138.3, 130.9, 130.3, 129.9 (q, *J =* 31.6 Hz), 129.8, 124.4 (q, *J =* 3.7 Hz), 124.3 (q, *J =* 272.5 Hz), 123.2 (d, *J =* 3.7 Hz), 123.2, 119.9, 118.9.

#### 2.2.7. 2-Hydrazino-N-(3′-methoxybiphenyl-4-yl)-2-thioxoacetamide (3g)

Yield 0.72 g (54%), m.p. 131–131.5°С. ^1^H NMR (300 MHz, DMSO) *δ* 10.31 (br.s, 1H), 7.86 (d, *J =* 8.6 Hz, 2H), 7.74–7.60 (m, 3H), 7.41–7.30 (m, 1H), 7.28–7.14 (m, 3H), 6.95–6.87 (m, 1H), 3.82 (s, 3H); ^13^C NMR (75 MHz, DMSO) *δ* 167.2, 160.2, 158.4, 141.4, 137.0 (q, *J =* 72.4 Hz), 130.5, 127.6, 120.9, 120.1, 119.2, 113.4, 112.3, 112.2, 30.0.

#### 2.2.8. 2-Hydrazino-N-(4′-methylbiphenyl-3-yl)-2-thioxoacetamide (3h)

Yield 0.73 g (58%), m.p. 154–155°С. ^1^H NMR (300 MHz, DMSO) *δ* 10.30 (br.s, 1H), 8.04 (br.s, 1H), 7.80–7.74 (m, 1H), 7.57 (d, *J =* 8.1 Hz, 2H), 7.45–7.40 (m, 2H), 7.28 (d, *J =* 8.0 Hz, 2H), 2.35 (s, 3H); ^13^C NMR (75 MHz, DMSO) *δ* 167.4, 158.1, 140.7, 138.1, 137.1, 136.9, 129.6, 129.4, 126.5, 122.6, 118.8, 118.3, 20.7.

### 2.3. General procedure 2: preparation of compounds 3a–h (exemplified for compound 2a)

To *N*-(4′-chlorobiphenyl-3-yl)-2-hydrazine-2-thioxoacetamide (**3a**) (0.4 mmol, 0.12 g) was added succinic anhydride (0.5 mmol, 0.05 g) and acetic acid (4.0 µmol, 2.40 ml). The reaction mixture was heated at a temperature of 100 °C for 5 h. The progress of the reaction was monitored using thin-layer chromatography and 2% methanol in chloroform as eluent. The reaction mixture was cooled to room temperature and added water (40 ml). The formed precipitate was filtered and dried *in vacuo*.

#### 2.3.1. 3-(5-{[(4′-Chlorobiphenyl-3-yl)amino]carbonyl}-1,3,4-thiadiazol-2-yl)propanoic acid (2a, LK01373)

Yield 0.17 g (91%), m.p. 186–187°С. ^1^H NMR (300 MHz, DMSO) *δ* 11.20 (s, 1H), 8.18–8.16 (m, 1H), 7.89–7.83 (m, 1H), 7.70–7.65 (m, 2H), 7.58–7.52 (m, 2H), 7.49–7.46 (m, 2H), 3.39 (t, *J =* 7.0 Hz, 2H), 2.83 (t, *J =* 7.0 Hz, 2H); ^13^C NMR (75 MHz, DMSO) *δ* 174.0, 173.2, 165.9, 156.6, 139.5, 138.8, 138.5, 132.7, 129.7, 129.2, 128.6, 123.1, 120.3, 119.2, 32.8, 25.4; HRMS (ESI) *m/z* calcd. for С_18_H_14_ClN_2_NaO_3_S [M + Na^+^] 410.0342 Da, found 410.0337 ± 0.0020 Da.

#### 2.3.2. 3-(5-{[(4′-Chlorobiphenyl-4-yl)amino]carbonyl}-1,3,4-thiadiazol-2-yl)propanoic acid (2b, LK01274)

The title compound was synthesised analogously to **2a** using *N*-(4′-chlorobiphenyl-4-yl)-2-hydrazino-2-thioxoacetamide (**3b**). Yield 0.17 g (90%), m.p. 249–249.5°С. ^1^H NMR (300 MHz, DMSO) *δ* 11.24 (s, 1H), 7.97–7.92 (m, 2H), 7.74–7.68 (m, 4H), 7.53–7.48 (m, 2H), 3.39 (t, *J =* 7.0 Hz, 2H), 2.83 (t, *J =* 7.0 Hz, 2H); ^13^C NMR (75 MHz, DMSO) *δ* 173.6, 172.7, 165.6, 156.3, 138.3, 137.4, 134.9, 132.2, 128.8, 128.1, 126.8, 121.1, 32.7, 25.2; HRMS (ESI) *m/z* calcd. for С_18_H_15_ClN_3_O_3_S [M + H^+^] 388.0523 Da, found 388.0471 ± 0.0020 Da.

#### 2.3.3. 3-[5-({[4′-(Trifluoromethoxy)biphenyl-3-yl]amino}carbonyl)-1,3,4-thiadiazol-2-yl]propanoic acid (2c, LK01375)

The title compound was synthesised analogously to **2a** using 2-hydrazino-2-thioxo-*N*-[4′-(trifluoromethoxy)biphenyl-3-yl]acetamide (**3c**). Yield 0.17 g (92%), m.p. 196–197°С. ^1^H NMR (300 MHz, DMSO) *δ* 11.22 (s, 1H), 8.19–8.17 (m, 1H), 7.90–7.85 (m, 1H), 7.79–7.75 (m, 2H), 7.51–7.47 (m, 4H), 3.39 (t, *J =* 7.0 Hz, 2H), 2.83 (t, *J =* 7.0 Hz, 2H); ^13^C NMR (75 MHz, DMSO) *δ* 173.9, 173.1, 165.9, 156.6, 148.1, 139.4, 139.3, 138.4, 129.7, 128.7, 123.2, 121.7, 120.3, 120.2 (q, *J =* 256.9 Hz), 119.3, 32.8, 25.4; HRMS (ESI) *m/z* calcd. for С_19_H_15_F_3_N_3_O_4_S [M + H^+^] 438.0735 Da, found 438.0730 ± 0.0020 Da.

#### 2.3.4. 3-(5-{[(4′-Methoxybiphenyl-4-yl)amino]carbonyl}-1,3,4-thiadiazol-2-yl)propanoic acid (2d, LK01379)

The title compound was synthesised analogously to **2a** using 2-hydrazino-*N*-(4′-methoxybiphenyl-4-yl)-2-thioxoacetamide (**3d**). Yield 0.19 g (86%), m.p. 155–156°С. ^1^H NMR (300 MHz, DMSO) *δ* 12.39 (br.s, 1H), 11.14 (s, 1H), 7.90 (d, *J =* 8.6 Hz, 2H), 7.65–7.59 (m, 4H), 7.02 (d, *J =* 8.6 Hz, 2H), 3.80 (s, 3H), 3.40 (t, *J =* 6.9 Hz, 2H), 2.83 (t, *J =* 6.9 Hz, 2H); ^13^C NMR (75 MHz, DMSO) *δ* 173.7, 173.1, 166.0, 158.8, 156.3, 136.6, 136.1, 132.0, 127.6, 126.4, 121.2, 114.4, 55.22, 32.8, 25.3; HRMS (ESI) *m/z* calcd. for С_19_H_19_N_3_O_4_S [M + H^+^] 384.1018 Da, found 384.1013 ± 0.0020 Da.

#### 2.3.5. 3-[5-({[3′-(Trifluoromethyl)biphenyl-4-yl]amino}carbonyl)-1,3,4-thiadiazol-2-yl]propanoic acid (2e, LK01380)

The title compound was synthesised analogously to **2a** using 2-hydrazino-2-thioxo-*N*-[3′-(trifluoromethyl)biphenyl-4-yl]acetamide (**3e**). Yield 0.18 g (84%), m.p. 209–210°С. ^1^H NMR (300 MHz, DMSO) *δ* 11.23 (s, 1H), 8.03–7.96 (m, 4H), 7.79 (d, *J =* 8.8 Hz, 2H), 7.72–7.68 (m, 2H), 3.40 (t, *J =* 6.9 Hz, 2H), 2.83 (t, *J =* 6.9 Hz, 2H); ^13^C NMR (75 MHz, DMSO) *δ* 173.8, 173.1, 165.8, 156.5, 140.6, 138.0, 134.6, 130.6, 130.1, 129.9 (q, *J =* 31.6 Hz), 127.4, 124.3 (q, *J =* 272.5 Hz), 123.9 (d, *J =* 3.4 Hz), 122.8 (q, *J =* 3.8 Hz), 121.2, 32.8, 25.3; HRMS (ESI) *m/z* calcd. for С_19_H_15_F_3_N_3_O_3_S [M + H^+^] 422.0786 Da, found 422.0781 ± 0.0020 Da.

#### 2.3.6. 3-[5-({[3′-(Trifluoromethyl)biphenyl-3-yl]amino}carbonyl)-1,3,4-thiadiazol-2-yl]propanoic acid (2f, LK01381)

The title compound was synthesised analogously to **2a** using 2-hydrazino-2-thioxo-*N*-[3′-(trifluoromethyl)biphenyl-3-yl]acetamide (**3f**). Yield 0.15 g (93%), m.p. 159–160°С. ^1^H NMR (300 MHz, DMSO) *δ* 12.24 (br.s, 1H), 11.06 (s, 1H), 8.23–8.19 (m, 1H), 8.01–7.92 (m, 3H), 7.77–7.72 (m, 2H), 7.58–7.48 (m, 2H), 3.41 (t, *J =* 7.0 Hz, 2H), 2.84 (t, *J =* 7.0 Hz, 2H); ^13^C NMR (75 MHz, DMSO) *δ* 173.9, 173.0, 165.8, 156.5, 140.9, 139.0, 138.5, 130.8, 130.3, 129.9 (q, *J =* 31.6 Hz), 129.7, 124.4 (q, *J =* 3.6 Hz), 124.3 (q, *J =* 272.4 Hz), 123.2, 123.0 (q, *J =* 3.8 Hz), 120.5, 119.3, 32.8, 25.3; HRMS (ESI) *m/z* calcd. for С_19_H_15_F_3_N_3_O_3_S [M + H^+^] 422.0786 Da, found 422.0781 ± 0.0020 Da.

#### 2.3.7. 3-(5-{[(3′-Methoxybiphenyl-4-yl)amino]carbonyl}-1,3,4-thiadiazol-2-yl)propanoic acid (2g, LK01384)

The title compound was synthesised analogously to **2a** using 2-hydrazino-*N*-(3′-methoxybiphenyl-4-yl)-2-thioxoacetamide (**3g**). Yield 0.13 g (76%), m.p. 204–204.5°С. 1H NMR (300 MHz, DMSO) *δ* 11.19 (s, 1H), 7.93 (d, *J =* 8.6 Hz, 2H), 7.70 (d, *J =* 8.7 Hz, 2H), 7.37 (t, *J =* 7.8 Hz, 1H), 7.26–7.19 (m, 2H), 6.94–6.90 (m, 1H), 3.83 (s, 3H), 3.40 (t, *J =* 7.0 Hz, 2H), 2.83 (t, *J =* 7.0 Hz, 2H); ^13^C NMR (75 MHz, DMSO) *δ* 173.9, 173.2, 166.0, 159.9, 156.5, 141.1, 137.4, 136.3, 130.2, 127.2, 121.2, 118.9, 113.0, 112.0, 55.2, 32.8, 25.4; HRMS (ESI) *m/z* calcd. for С_19_H_17_N_3_NaO_4_S [M + Na^+^] 406.0837 Da, found 406.0832 ± 0.0020 Da.

#### 2.3.8. 3-(5-{[(4′-Methylbiphenyl-3-yl)amino]carbonyl}-1,3,4-thiadiazol-2-yl)propanoic acid (2h, LK01397)

The title compound was synthesised analogously to **2a** using 2-hydrazino-*N*-(4′-methylbiphenyl-3-yl)-2-thioxoacetamide (**3h**). Yield 0.11 g (77%), m.p. 192–193°С. ^1^H NMR (300 MHz, DMSO) *δ* 11.17 (s, 1H), 8.16–8.13 (m, 1H), 7.85–7.80 (m, 1H), 7.56–7.53 (m, 2H), 7.46–7.43 (m, 2H), 7.30 (d, *J =* 7.9 Hz, 2H), 3.39 (t, *J =* 7.0 Hz, 2H), 2.81 (t, *J =* 7.0 Hz, 2H), 2.35 (s, 3H); ^13^C NMR (75 MHz, DMSO) *δ* 174.0, 172.9, 165.8, 156.4, 140.6, 138.2, 137.0, 134.7, 129.6, 129.3, 126.4, 122.7, 119.5, 118.9, 33.2, 25.5, 20.6; HRMS (ESI) *m/z* calcd. for С_19_H_18_N_3_O_3_S [M + H^+^] 368.1069 Da, found 368.1063 ± 0.0020 Da.

### 2.4. *In vitro* FFA1 activation assay

CHO cells stably expressing human FFA1 (stable CHO-GPR40 line created at Enamine Ltd.) were seeded (12,500 cells/well) into 384-well black-wall, clear-bottom microtiter plates 24 h prior to assay. Cells were loaded for 1 h with fluorescent calcium dye (Fluo-8 Calcium Assay kit, Abcam, ab112129) and tested using fluorometric imaging plate reader (FLIPR Tetra^®^ High Throughput Cellular Screening System, Molecular Devices Corp.). The maximum change in fluorescence over the base line was used to determine agonist response. A potent and selective agonist for FFA1 GW9508 (Selleckchem, S8014) was tested with the test compounds as a positive control. Concentration-response curve data were fitted using Molecular Devices ScreenWorks^®^ System Control Software (Molecular Devices).

### 2.5. *In silico* studies

#### 2.5.1. Protein structure preparation

The structure of FFA1 (GPR40) was obtained from RCSB Protein Data Bank (4PHU) and prepared for *in silico* modelling using Protein Prepwizard. Incorrectly defined chemical bonds were corrected, missing amino acid side chains were reconstructed, crystal water was removed, the hydrogen bond network was optimised, and limited minimisation of the model was performed^9^.

The protein model was supplemented with a structure of the lipid cell membrane bilayer assembled from phosphatidyl ethanolamine (POPE) The membrane was positioned relative to the protein-based on the information from the library of transmembrane proteins OPM^10^. The following transmembrane segment information was used: 1 (5–28), 2 (43–67), 3 (76–102), 4 (123–144), 5 (179–204), 6 (2223–2249), 7 (2257–2276). The membrane structure was generated using the toolbox of the Schrödinger Suite 2020–4.

#### 2.5.2. Small molecule preparation

The three-dimensional coordinates of all small molecules were generated in the OPLS3e force field using the LigPrep module of the Schrödinger Suite 2020–4, aiming at the lowest energy conformations taking into account stereoisomerism (if applicable) and tautomerism (if it exists).

#### 2.5.3. Docking procedure

The compounds were docked into the active site of FFA1 (GPR40) using the Glide module^11^, in the standard precision mode (SP). The area for docking was defined based on the positioning of the ligand in the crystal structure (4PHU). For each compound, up to 20 docking poses were generated. The best poses were selected based on the optimal reproduction of the native ligand binding by the ligand in question as well as based on the clustering of the docking solutions (RMSD ≤ 2.2 Å within each cluster).

#### 2.5.4. Strain analysis in the ligand–protein complex

For the resulting ligand–protein complexes, free energy (ΔG) components were calculated using MM-GBSA method^12^. The most important parameter in the context of this work – strain energy of the ligand–protein complexes (ΔG^strain^, kcal/mol). The calculations took into account the presence of solvent (water).

## 3. Results and discussion

### 3.1. Chemistry

**Scheme 1.**
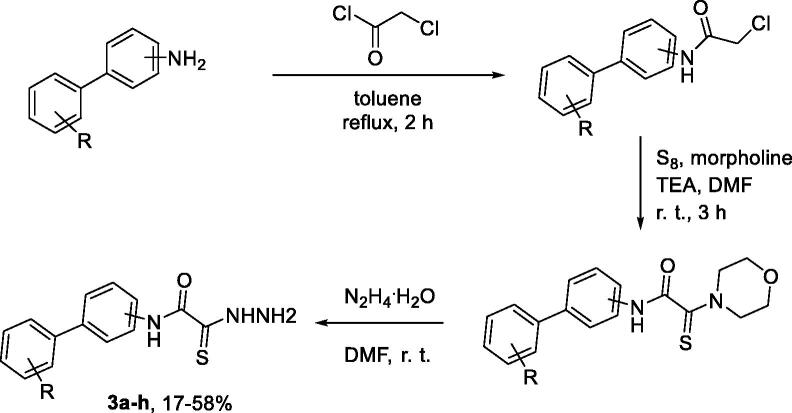
Synthesis of thiohydrazides **3a–h**.

The starting thiohydrazides **3a–h** were synthesised in three chemical operations from substituted biphenylamines. Acylation with chloroacetyl chloride followed by Willgerodt–Kindler-type reaction^13^ and treatment with hydrazine hydrate furnished the desired thiohydrazides **3a–h** in moderate to good yield over three steps (Scheme 1).

**Scheme 2.**
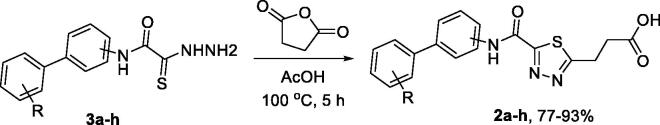
Synthesis of 1,3,4-thiadiazoles **2a–h**.

Thiohydrazides **3a–h** thus obtained were converted in good to excellent yield to 1,3,4-thiadiazoles **2a–h** on reaction with succinic anhydride in acetic acid performed over 5h at near-reflux temperature (Scheme 2).

### 3.2. Biological evaluation

1,3,4-thiadiazoles **2a–h** synthesised as described before were tested for their ability to activate FFA1 using calcium flux assay employing Chinese hamster ovary (CHO) cells engineered to stably express human FFA1. All compounds were first tested at 5 µM concentration to establish % FFA1 activation relative to commercially available reference FFA1 agonist GW9508^14^. Compounds displaying >50% FFA1 activation at that concentration relative to GW9508 were then tested in a concentration-response mode in order to calculate EC_50_ values. These data are summarised in Table 1.

**Table 1.** Agonistic potency of compounds **2a–h** against FFA1.

Compound	Code	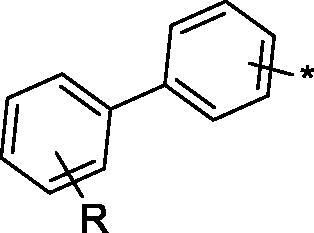	% FFA1 activation at 5 µM (relative to GW9508)	EC_50_, µM
**2a**	LK01373	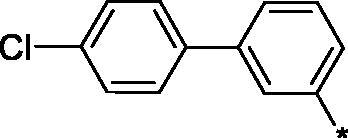	86	1.32
**2b**	LK01374	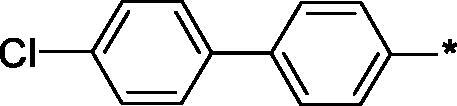	23	ND
**2c**	LK01375	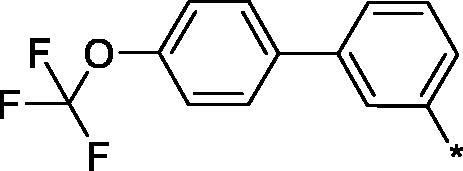	67	1.25
**2d**	LK01379	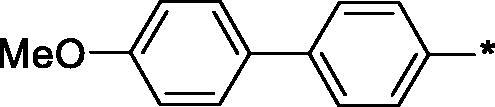	15	ND
**2e**	LK01380	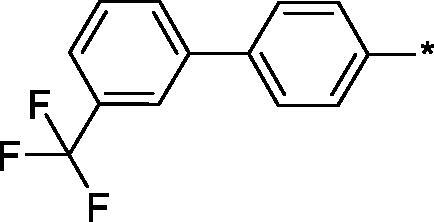	60	1.86
**2f**	LK01381	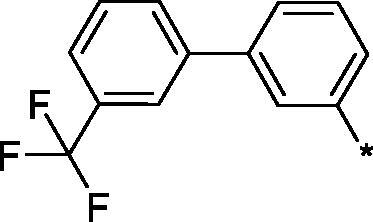	43	ND
**2g**	LK01384	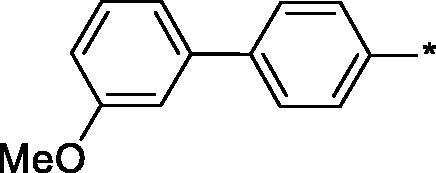	66	1.35
**2h**	LK01397	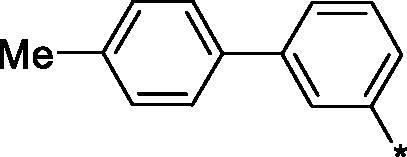	19	ND

ND: not determined; GW9508 EC_50_ 0.047 µM^14^.

As it follows from the data presented in Table 1, the four active FFA1 agonists (**2a**, **2c**, **2e**, and **2g**) still reside in the low-µM range of potency. Attempts to draw any SAR generalisations were unsuccessful. For example, it was not clear why the difference in activity of structurally close **2a** or **2c** and **2h** was so dramatic. Hence, as stated in the Introduction, we turned our attention to docking these agonists into the crystal structure of FFA1 built in the model of phospholipid cell membrane bilayer in order to determine whether any strained contacts between the compounds tested and the membrane could help rationalise the observed activity trends.

### 3.3. Docking studies

**Figure 2.**
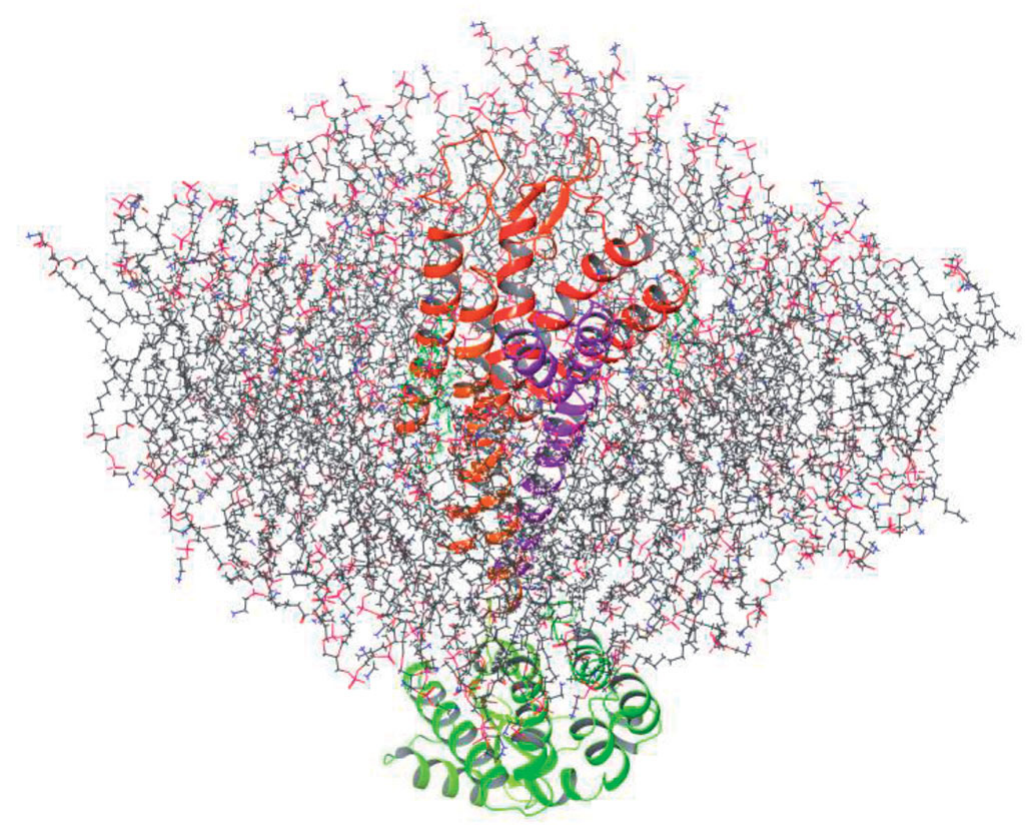
Model of FFA1 built in the phospholipid cell membrane bilayer.

The model of FFA1 built in the phospholipid cell membrane bilayer was constructed as described in Materials and methods (Figure 2).

**Figure 3.**
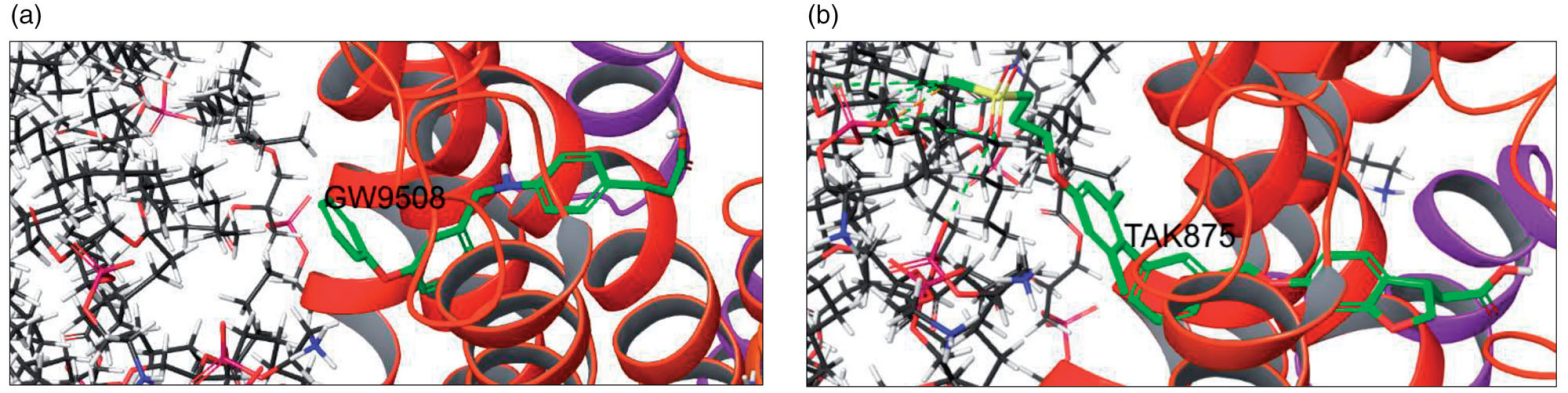
Docking of (a) GW9508 and (b) TAK875 into the FFA1-cell membrane model (no contacts for (a) and non-strained contacts (green dotted line) for (b)).

Docking of the reference structures of potent FFA1 agonists GW9508 and TAK875 revealed that they display either no contacts with the cell membrane (GW9508) or these contacts are not strained (TAK875) (Figure 3).

**Figure 4.**
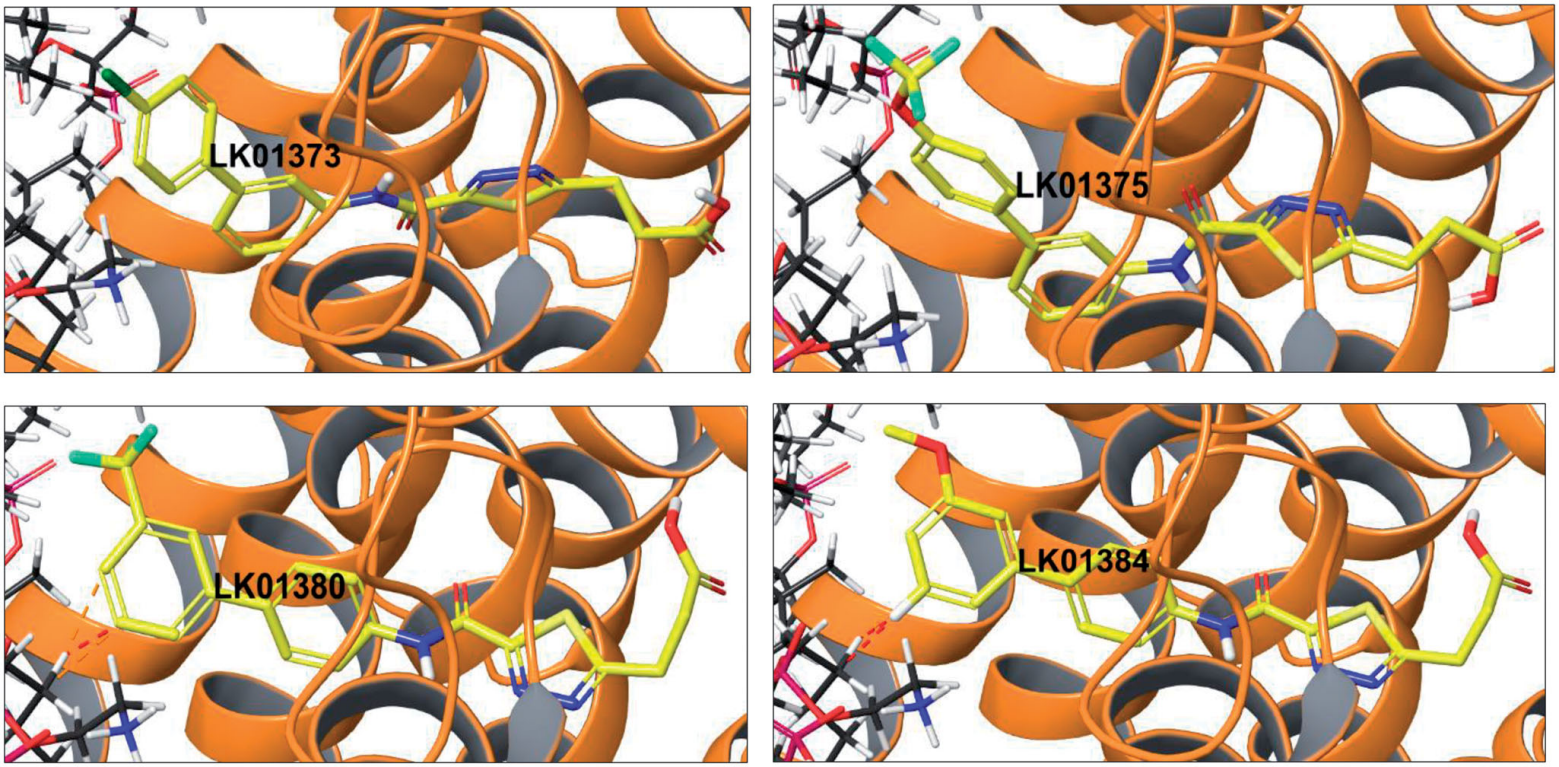
Docking pose of active compounds **2a** (LK01373), **2c** (LK1375), **2e** (LK1380), and **2g** (LK01384) displaying little or no strained contacts with the cell membrane.

**Figure 5.**
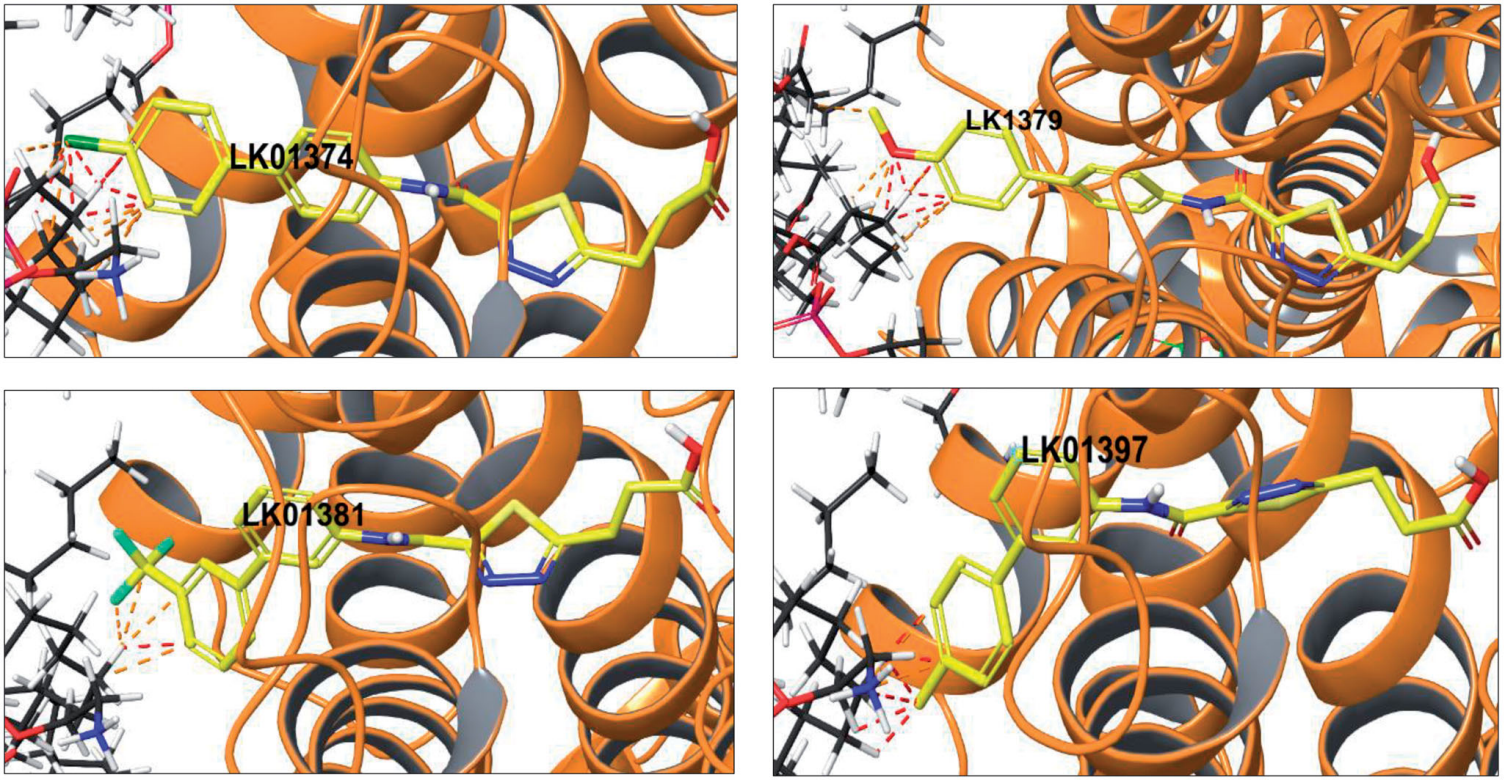
Docking pose of active compounds **2b** (LK01374), **2d** (LK1379), **2f** (LK1381), and **2h** (LK01397) displaying many strained contacts with the cell membrane.

When a similar assessment of ligand contacts with the cell membrane was performed for four active (**2a**, **2c**, **2e**, and **2g**) and four inactive, albeit structurally related compounds (**2b**, **2d**, **2f**, and **2h**), it was established that the active cohort displayed little strained or only favourable (i.e. non-strained) contacts with the membrane (Figure 4). At the same time, the inactive cohort displayed numerous strained contacts with the membrane (Figure 5).

The apparent influence of the free energy of strained contacts with the cell membrane (ΔG^strain^) – and not the GlideScore obtained by docking compounds into the isolated FFA1 structure (which is not drastically different for the active and inactive cohort) – on the agonistic potency of compounds **2a–h** is illustrated by the respective values summarised in Table 2.

**Table 2.** GlideScore and ΔG^strain^ values for compounds **2a–h** vs. their activity towards FFA1.

Compound	Code	% FFA1 activation relative to GW9508 (5 µM)	EC_50_, µM	GlideScore	ΔG^strain^
**2a**	LK01373	86	1.32	−8.48	2.39
**2b**	LK01374	23		−7.06	**10.51**
**2c**	LK01375	67	1.25	−8.28	3.30
**2d**	LK01379	15		−6.69	**10.57**
**2e**	LK01380	60	1.86	−8.72	4.54
**2f**	LK01381	43		−8.45	**5.98**
**2g**	LK01384	66	1.35	−8.43	3.49
**2h**	LK01397	19		−7.76	**8.39**
GW9508		100	0.047	−9.28	1.01

Bold values signify the most strained energy.

## 4. Conclusions

We have demonstrated that docking with Glide SP into the available crystal structure of a G protein-coupled receptor and the resulting docking scores can be insufficient to rationalise the observed activity trends. By reconstructing the phospholipid cell membrane bilayer around the free fatty receptor 1 we were able to assess the energy of strained contacts of structurally related ligands with the cell membrane. It was established that while active and inactive compounds did not differ significantly in docking scores, the inactive cohort displayed significant values of unfavourable strain energy which is the likely reason for the observed absence of functional activity of these compounds with respect to the receptor.

Detailed experimental procedures for synthesis, complete characterisation data, and copies of ^1^H and ^13^C NMR spectra are available online.
